# The Hospital Problem

**Published:** 1892-04-16

**Authors:** 


### The Hospital

An Institutional Journal of

Science, Medicine, Nursing, and Philanthropy.

*For Hospitals, Asylums, Infirmaries, Dispensaries, Medical Practitioners, Students, Nurses, and the Charitable Public.*

Vol. XII.—No. 290.] [April 16, 1892.

### THE HOSPITAL PROBLEM.

We are happy to be able to announce that the report of the Lords' Committee on the metropolitan hospitals is now printed, and that it will be distributed about Easter. It is hoped that the report will contain suggestions of so practical a character as to be capable of easy adoption. It is now generally agreed that advantage would accrue to the hospitals as well as to the public if some central board or authority representative of all the hospitals could be set up. Its functions would be to educate and guide public opinion, to direct the free gifts of the public to hospitals into the proper channels, and to arrange that the system of medical relief should be more equally distributed over the whole of the metropolis. A recent correspondent wisely declares that the hospital patient is a creature *sui generis.* He comes to the Westminster Hospital all the way from Mile End, and goes from Kensington to St. Bartholomew's Hospital. No doubt these erratic wanderings would continue despite all attempts to control them, but nothing but good should result from the establishment of a representative authority so constituted as to secure the cordial co-operation and support of the whole of the reputable hospitals. No doubt changes in the direction indicated would tend to remove some evils, and to lessen the difficulties which at present beset the most active hospital managers in London. Every year it becomes more apparent that to go on in the old groove, issuing appeals by the thousand, nine-tenths, and probably ninety-nine hundredths of which go into the waste paper basket, is, to say the least inartistic, irritating to the most devoted of secretaries, and inadequate when tested by results, so far as the hospital exchequer is concerned. The greater the experience of the hospital secretary, the more he is beset with doubts as to the probable success of any attempt whatever to move the slough of despond which is typified in the great mass of people who at present give nothing to the hospitals. There must, of course, always be a deficit so far as the voluntary hospitals are concerned, but we agree with those who contend that it must never be allowed to assume a chronic condition, despite the fact that the most effective plea is often found to be the unbearable burden of debt which weighs down a particular committee, and threatens to paralyse their efforts on behalf of the sick and suffering. We are convinced from a careful consideration of all the facts and a knowledge of the actual amounts given in charity in London every year, that plenty of money is forthcoming if the hospitals could only set up a directing hand which would fix public attention, and so secure that an adequate proportion of the gifts of the benevolent should find their way into the hospital chest. Whether the Lords' Committee will be prepared to suggest some plan by which this may be accomplished remains to be seen. In any case the need exists, and the sooner an attempt is made to supply it the better.

We have ascertained that the metropolitan hospitals as represented by those institutions which receive grants from the Hospital Sunday Fund, had to raise during 1891 nearly a quarter of a million sterling in benefactions over and above their reliable income. It is very difficult to define what should be classed as reliable income, but we think most people will agree that under this term should be included (1) rents, dividends and interest on investments of every description; (2) annual subscriptions; (3) grants from the Hospital Sunday and Saturday Funds; and (4) payments by patients; while legacies and donations cannot rightly be classed as reliable. Now this deficiency of £250,000 is a singular fact, and it is most encouraging that the secretaries should have united after due deliberation in an attempt to awaken the public conscience, and so securing that henceforward the income of the metropolitan hospitals should bear a much larger proportion to the necessary annual expenditure. These gentlemen have appointed a committee, which has been at work for some months, and the results of its labours will be seen soon after Easter. Meanwhile, it is lamentable to see that this cordial attempt at co-operation on behalf of all the hospitals in the interests of each individual institution has been assailed by a few interested persons, who have heretofore profited by the absence of combination. Such criticism bears the imprint of personal motive for selfish ends, and it is satisfactory to note that the only effect it is likely to have is to strengthen the feeling of cordiality which has produced the present co-operation amongst hospital secretaries, and so to permanently benefit the whole of the metropolitan hospitals.

"General" Booth gave the hospitals an object lesson last year which should not fail to carry a moral with it. It is true that there is a general feeling that all the facts about hospitals are known, and that nothing new can be said on the subject. This is a very superficial view to take of a great question, and we hope and believe that within the next two months circumstances will show its erroneous character, and that at last the public conscience will be aroused, and so the vast majority of the citizens in London will follow the example of the citizens of provincial towns and come forward in such numbers as to secure not only the adequate support of the hospitals, but an abundance of willing and capable spirits who will give not only money but personal service in so noble a cause. Many people have contended that the working classes have come to regard all sickness as a matter for relief from parish or charity, that they have therefore no duty and little if any responsibility so far as the hospitals are concerned. This statement is, however, disproved by the notable increase in the support which has been extended to the Metropolitan Hospital Saturday Fund since Mr. Acland and those who have so actively co-operated with him have set themselves to reorganise and extend this excellent movement amongst the artizans and workpeople of London.

It is unjust for middle class subscribers to contend, as some of them do, that as there is no demand for the tickets which they receive in return for their subscriptions they see no use in continuing them. Surely anyone who subscribes to the hospitals gives his money because he believes in the soundness of the cause which is represented by the relief of the sick and suffering poor. If that be so, not only should a subscriber not grumble when his tickets remain unutilised, but in those years when they are utilised he should take care to increase his gift to the hospitals, so as to secure that the sum represented by the subscription when the tickets are not used is always the minimum sum which he expends in the charity which the hospital represents so far as expenditure is concerned.

At any rate grumblers, few though they be, such as those we refer to, cannot with any fairness turn round on the working man and say it is your duty to support the hospitals because they are used by yourselves and your families. The working classes have always shown an excellent spirit in regard to the hospitals, and they will, we are certain, go forward under Mr. Acland's auspices in London and raise the Hospital Saturday Fund to the position of importance it undoubtedly ought to occupy. In any case the present is not a time for criticism, seeing that the needs of the hospitals were never greater than they are at this moment, and so every intelligent man and woman who is also a good citizen of this vast city, should cheerfully combine with the object of relieving the present pressure in regard to funds and of placing the voluntary hospitals of London once and for all upon a sound financial footing.

